# PLACE MATTERS: HOW LOCATION AT DEATH INFLUENCES CAREGIVER WELL-BEING IN BEREAVEMENT

**DOI:** 10.1093/geroni/igz038.495

**Published:** 2019-11-08

**Authors:** Deborah P Waldrop, Jacqueline M McGinley

**Affiliations:** 1 University at Buffalo School of Social Work, Buffalo, New York, United States; 2 Buffalo State College, Buffalo, New York, United States

## Abstract

Most older adults express the preference to die at home, but the desire for home death may go unfulfilled when the dying process become burdensome. Little is known about the congruence between older adults’ and their caregivers’ desired locations at death. The purpose of this study was to explore how the congruence between caregiver-care recipients desired and actual location at death influenced well-being in bereavement. This exploratory study utilized simultaneous qualitative and quantitative methods. Interviews were conducted with 108 bereaved caregivers about 4 months after the care recipient died while receiving hospice care. Care recipients’ ages ranged from 43-101 (M=79.6); caregivers from 32-88 (M=61.5). Quantitative data included categorical variables about demographics, advance care planning and location at death. The Core Bereavement Items and CDC HRQOL–14 "Healthy Days Measure" scales were used. Qualitative data involved open-ended questions about the illness trajectory, desired location and perceptions of care at life’s end. Quantitative analysis included comparison of group differences using both Independent Samples t-tests and One-way ANOVA. Of the 92 care recipients who had an advance directive, N=49 (45%) were in the location they desired and for N=49 (45%) there was caregiver/care recipient congruence about location. Caregivers who experienced incongruence reported poorer physical and emotional well-being and higher, more intense bereavement symptoms. Three overarching themes illuminated caregivers’ experiences: (1) Caregiver-recipient congruence; (2) Caregiver-recipient incongruence; (3) Incongruence-influenced bereavement. Results suggest that incongruence between desired and actual location of death affects well-being in bereavement. Implications: Communication about location at death is an essential consideration.

